# *Pseudozyma aphidis* Suppresses Microbe-Associated Molecular Pattern (MAMP)-Triggered Callose Deposition and Can Penetrate Leaf Tissue

**DOI:** 10.1128/spectrum.02638-21

**Published:** 2022-03-02

**Authors:** Shanee Alster, Avis Dafa-Berger, Aviva Gafni, Maggie Levy

**Affiliations:** a Department of Plant Pathology and Microbiology, The Robert H. Smith Faculty of Agriculture, Food and Environment, The Hebrew University of Jerusalem, Rehovot, Israel; University of Molise

**Keywords:** biocontrol, callose deposition, endophyte, MAMPs, *Pseudozyma aphidis*

## Abstract

Beneficial microorganisms need to overcome the plant defense system to establish on or within plant tissues. Like pathogens, beneficial microbes can manipulate a plant’s immunity pathways, first by suppressing and hiding to establish on the host and then by inducing resistance to protect the plant. In the current study, we demonstrated that although Pseudozyma aphidis can activate microbe-associated molecular pattern (MAMP)-associated genes, it does not activate MAMP-triggered callose deposition and can, moreover, suppress such deposition triggered by Flg22 or chitin. While MAMP-associated gene activation by P. aphidis was not dependent on salicylic acid, jasmonic acid, or ethylene signaling, suppression of MAMP-triggered callose deposition required the salicylic acid and jasmonic acid signaling factors JAR1-1 and E3 ubiquitin ligase COI1 yet did not rely on EIN2, NPR1, or the transcription factor JIN1/MYC2. We also demonstrated the ability of *P. aphidis*, known to be an epiphytic yeast-like organism, to penetrate the stomata and establish within plant tissues, as do endophytes. These results thus demonstrate the potential of *P. aphidis* to suppress MAMP-elicited defenses in order to establish on and within host plant tissues.

**IMPORTANCE** Our study demonstrates the ability of *P. aphidis* to penetrate into plant tissues, where it avoids and overcomes plant defense systems in order to establish and subsequently protect the plant.

## INTRODUCTION

In the environment, plants encounter a variety of different microbes. Beneficial and pathogenic microbes that successfully invade a plant mostly have the ability to manipulate the plant’s immune system to reprogram the host response and metabolism. On the other hand, plants have developed a multilayered defense system that includes preexisting structural barriers, as well as induced immune defense. Induced defense depends on the recognition of conserved microbial structures known as microbe-associated molecular patterns (MAMPs), such as bacterial flagella, or major components of fungal cell walls, such as chitin (1, 2). MAMPs recognition can trigger downstream signaling that leads to the activation of a defense response known as MAMP-triggered immunity (MTI). MTI activates callose deposition, with callose frequently accumulating in the penetration zone as a physical barrier that can also activate defense signaling (3–5). Microbes that succeed in confronting or avoiding this layer of defense then encounter a second line of defense layer which is more efficient and specific, involves effector recognition, and is accordingly termed effector-triggered immunity (ETI). The induced defense response is regulated by a hormonal signaling network in which the plant hormones jasmonic acid (JA), salicylic acid (SA), ethylene (ET), and abscisic acid (ABA) play major roles (6–9). This signaling network determines the nature of the defense response to a specific pathogen or pest by activating a specific set of pathogenesis-related (PR) genes (8, 10–13). Systemic defense can be achieved by induction of the systemic acquired response (SAR), which is induced upon local contact with a pathogen and depends on the plant hormone SA and the NPR1 protein (14–16), as well as on the activation of PR gene expression (7, 17, 18), yet can also be SA-independent (19, 20, 21). Alternatively, induced systemic resistance (ISR) can be induced by beneficial microorganisms and depends mainly on the plant hormones JA and ET and the NPR1 protein but without the induction of PR genes (22–25). Many microorganisms and pathogens have evolved strategies to overcome the plant immune response, including injection of virulence effectors that suppress basal and induced defense responses (26, 27).

Fungal biocontrol agents have become an important alternative to the use of chemicals due to environmental concerns. Biological control can be achieved by one mechanism or a combination of mechanisms, such as antibiosis, mycoparasitism, competition, and induced resistance in the host plant. These mechanisms can hinder growth and development of the pathogen, thereby reducing disease. The complex mode of action of biocontrol agents obscures the ability of a pathogen to develop resistance. It is thought that biocontrol agents can protect plants from diverse pathogens by inducing systemic resistance mechanisms that are often associated with upregulation of PR genes and/or accumulation of phytoalexins (28–31). Trichoderma asperellum, for example, is an effective biocontrol agent for a number of soilborne pathogens of cucumber. Specifically, T. asperellum infection modulates the expression of genes involved in JA/ET-signaling pathways recruited for inducing systemic resistance (29). Similarly, in *Arabidopsis*, resistance to powdery mildew can be conferred by the mycorrhizal fungus Piriformospora indica through systemic resistance (30). P. indica induces resistance via JA signaling and NPR1 (30). Penicillium simplicissimum can induce resistance in *Arabidopsis* by activating multiple defense mechanisms, including both SA- and JA/ET-signaling pathways (31). Transcription analysis of plant interaction with Trichoderma hamatum failed to detect induction of ISR markers, with upregulation of only one marker of SAR (i.e., PR5) being seen (32). Therefore, it is likely that different biocontrol agents use different mechanisms to induce plant defense.

Epiphytic yeasts that colonize different plant surfaces (33, 34) are thought to possess biocontrol activity and provide a natural barrier against certain plant pathogens (35–40). Biocontrol activity of yeasts and yeast-like fungi has been demonstrated for postharvest diseases (41–46) and diseases in the greenhouse (47–49). *Pseudozyma* spp. are a small group of yeast related to the *Ustilaginales* (50). Pseudozyma rugulosa, Pseudozyma flocculosa, and *P. aphidis* exhibit biological activity against the different pathogens with which they are associated (51–57). In our previous work, we demonstrated that *P. aphidis* establishes and persists on the plant surface and can serve as biocontrol agent against Botrytis cinerea, acting via a complex mode of action. We demonstrated that *P. aphidis* can induce resistance in a JA/ET- and SA-independent manner and activate programmed cell death (PCD) and reactive oxygen species (ROS) accumulation in the pathogen (58–60). We could also demonstrate that *P. aphidis* could activate induced resistance in tomato plants against the bacterial canker pathogen Clavibacter michiganensis (61). Finally, we were also able to show that *P. aphidis* can dimorph from a yeast-like to a hyphal morphology in different biotic and abiotic conditions (62). For instance, while interacting with powdery mildew, *P. aphidis* hyphae coil around the pathogen hyphae and inhibit it through ecto-parasitism (62). In the current study, we demonstrated that *P. aphidis* can penetrate into the plant cell through open stomata and establish within plant tissues as endophyte. We also demonstrated the ability of *P. aphidis*, even though it is recognized by the host, to suppress the MAMP-triggered callose deposition that is partially dependent on SA and JA.

## RESULTS

### *P. aphidis* secretes cuticle- and cell wall-lytic enzymes.

**FIG 1:**
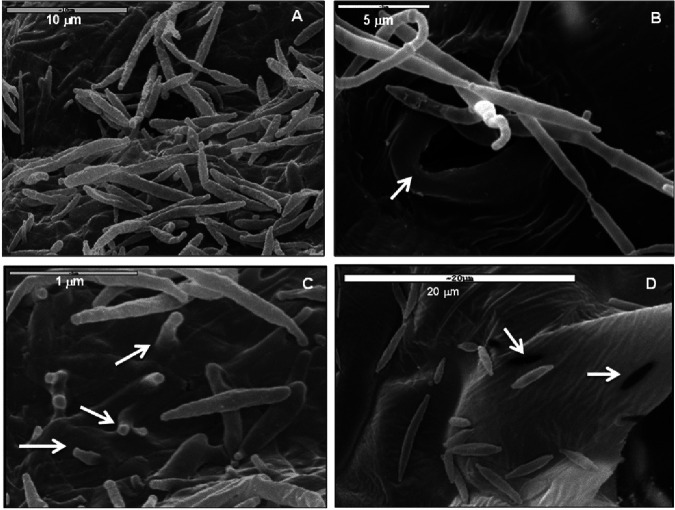
*P. aphidis* on the *Arabidopsis* surface. Scanning electron microscopy image of the surface of *Arabidopsis* treated with *P. aphidis*. (A) *P. aphidis* cells on the leaf surface. (B) *P. aphidis* cells in close proximity to a stomata opening (white arrow). (C) *P. aphidis* cells emerging from the cuticle back to the plant surface (denoted by a white arrow). (D) Footprinting of *P. aphidis* cells on the plant cuticle (denoted by a white arrow).

**FIG 2:**
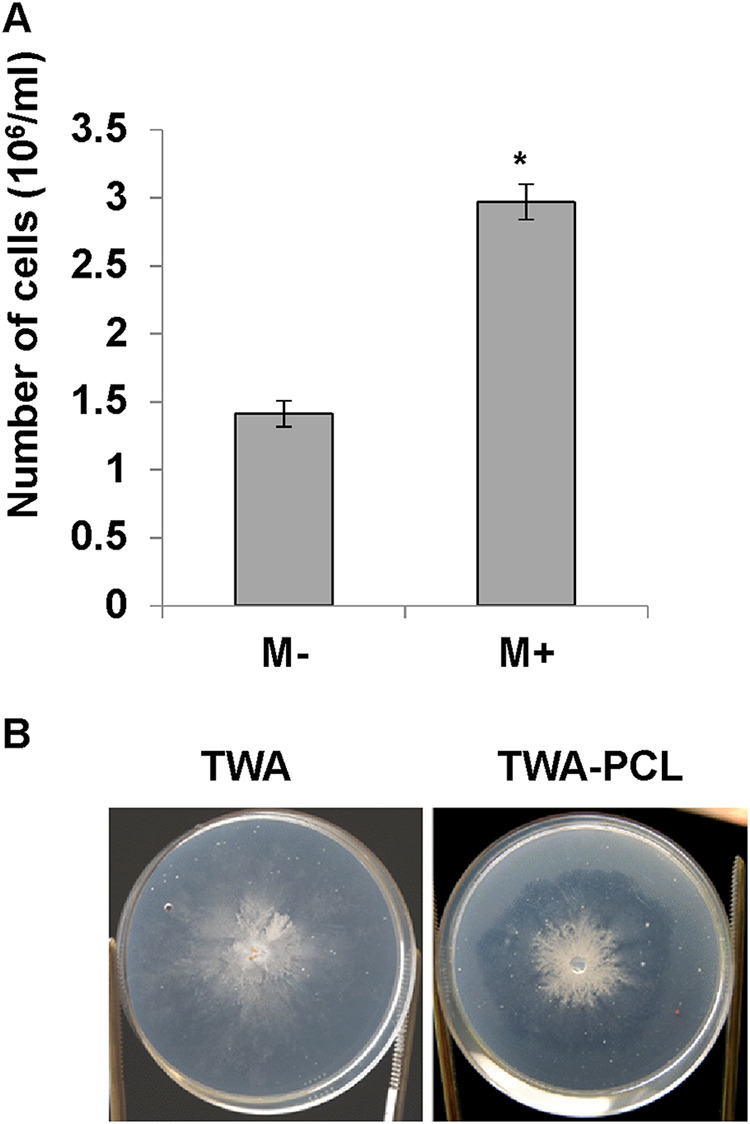
Cellulase and cutinase activity in *P. aphidis*. (A) *P. aphidis* grown on tap water agar (TWA) plates that were covered with a cellulose membrane (M+) or uncovered (M−). The number of cells on each plate was recorded 7 days after inoculation. Averages of 10 samples with standard error bars are presented. An asterisk indicates a significant difference as determined by *t* test (*P < *0.05). (B) *P. aphidis* grown on TWA supplemented with 1% PCL and TWA as a control.

*P. aphidis* is known as an epiphytic yeast-like fungus. Using scanning electron microscopy (SEM), we could demonstrate that *P. aphidis* cells are in close proximity to the cuticle plant surface and also to the stomata apparatuses (Fig. 1A and B). As indicated in Fig. 1C, we could also see that *P. aphidis* cells emerge through the cuticle to the surface, such that footprints of *P. aphidis* cells remain imprinted on the cuticle after the cells have moved from place to place (Fig. 1D). This could be indicative of some enzymatic activity occurring during establishment. To verify whether *P. aphidis* secretes enzymes that can degrade plant cell walls or cuticles, thereby helping it to establish onto the plant surface or penetrate the plant tissue, we grew *P. aphidis* on water agar plates supplemented with a cellulose membrane or polycaprolactone (PCL), a cutin analogue, as sole carbon source. In our previous work, we demonstrated by halo assay that *P. aphidis* secretes cellulase that degrades carboxymethylcellulose (60); here, we demonstrate in Fig. 2A that *P. aphidis* grew better on the cellulose membrane than on water agar (Fig. 2A). We also saw that *P. aphidis* secretes cutinase that degrades cutins added to the water agar plates, as demonstrated by the appearance of a halo around the colony (Fig. 2B). We did not, however, see excess growth on PCL, compared to controls (Fig. S1). These results suggest that *P. aphidis* can secrete cellulase and cutinase that degrade plant cell wall components to establish and penetrate into plant tissues.

### *P. aphidis* can penetrate plant tissue through stomata and establish as do endophytes.

**FIG 3:**
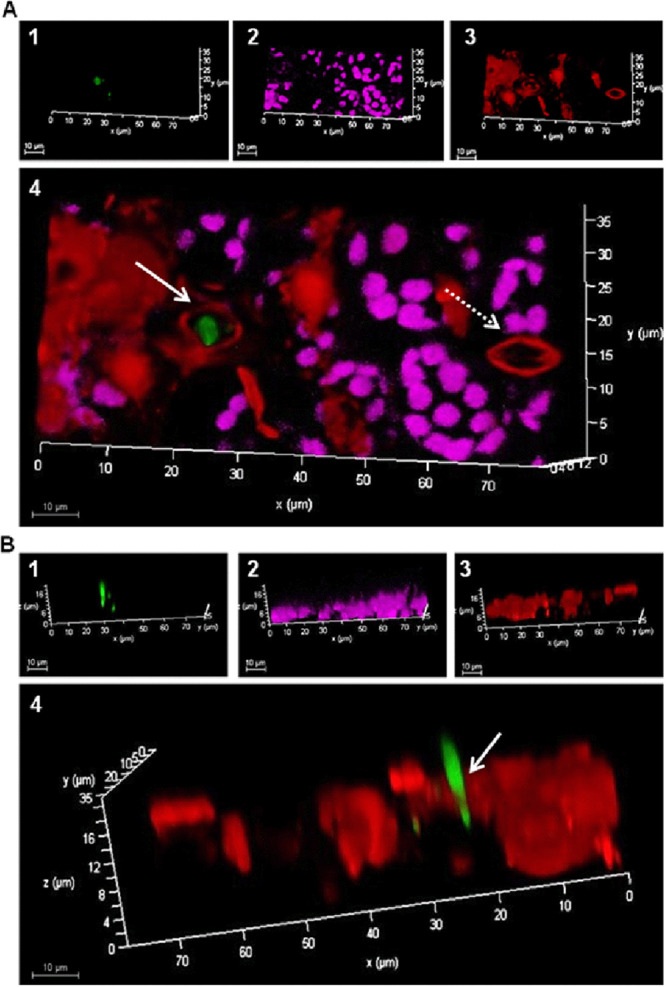
*P. aphidis* enter *Arabidopsis* plants through stomata. Confocal microscopy analysis of Arabidopsis thaliana leaves 3 days posttreatment with GFP-tagged *P. aphidis* (PA-GFP). (A and B) (1) PA-GFP channel, (2) autofluorescent channel for chloroplasts, and (3) propidium iodide stain (red) that outlines the epidermis and nuclei. (A4) A merged view of the plant leaf surface shows open stomata (red, marked with a solid arrow) and *P. aphidis* (green) inside the stomatal apparatus, compared to closed stomata (red, marked with a dashed arrow). (B4) PA-GFP (green) located inside the stomata viewed from a different angle. GFP (green) was excited using a 488 nm laser to yield maximal emission at 500 nm, chloroplasts (purple) were excited using a 488 nm laser to yield maximal emission at 700 nm, and propidium iodide stain (red) was excited using a 514 nm laser to yield maximal emission at 610 nm. We separated the green laser (488 nm) emission to help distinguish between chloroplasts and the PA-GFP isolate.

**FIG 4:**
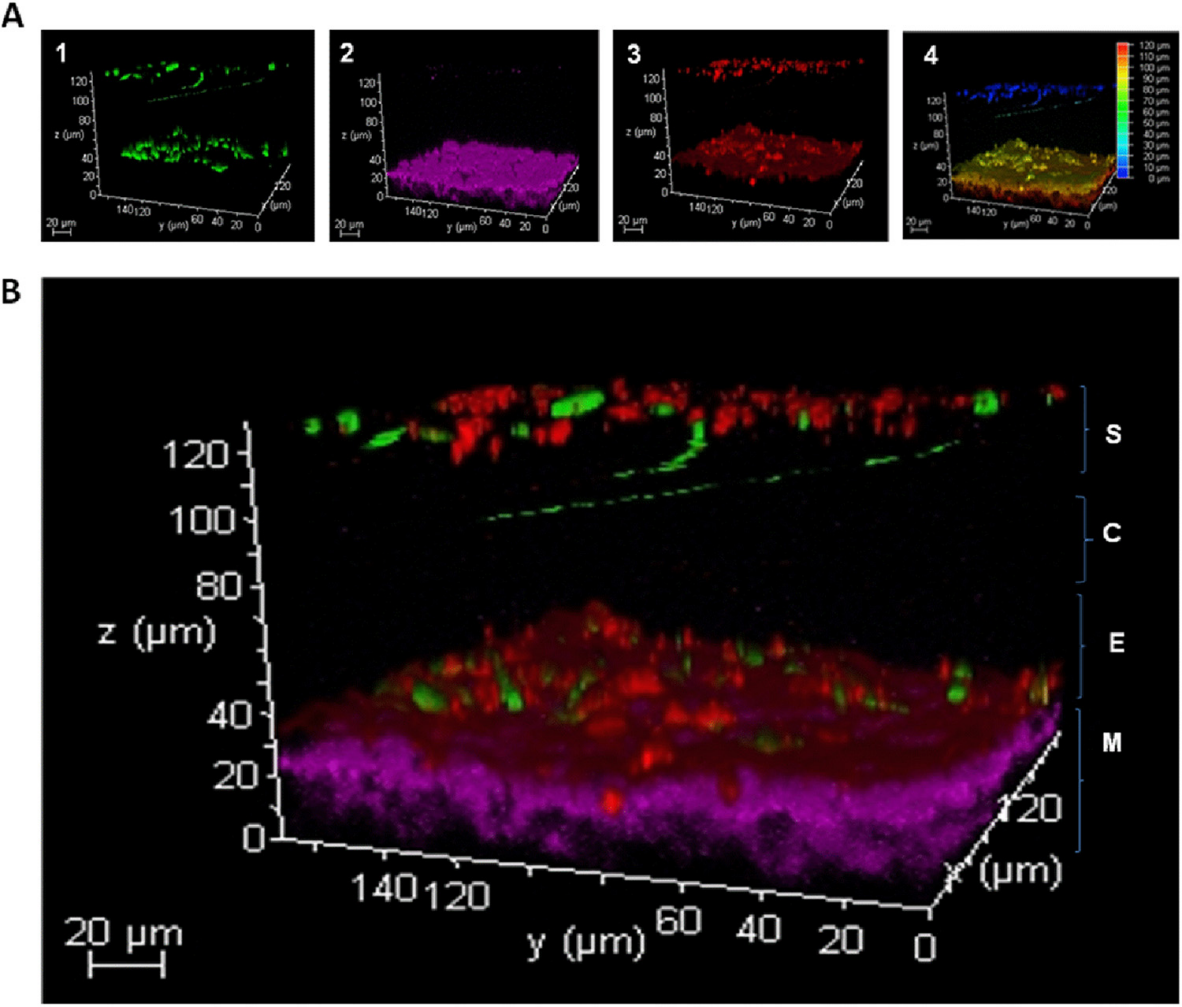
*P. aphidis* localization inside *Arabidopsis* cells. Confocal microscopy analysis of Arabidopsis thaliana leaves 3 days posttreatment with GFP-tagged *P. aphidis* (PA-GFP). (A) The following laser channels were used: (1) the PA-GFP channel, (2) the chloroplast autofluorescent channel, and (3) the propidium iodide stain channel (red) that outlines the epidermis and stains *P. aphidis* nuclei. (4) Different layers are marked according to a colored depth coding bar, showing the location of PA-GFP in the different dimensions according to depth layer (blue to red). The bar color reflects the direction of the start point of the Z stack layers. (B) The plant leaf Z stack merged view shows layers from the plant leaf surface (S), through the cuticle (C), covering the epidermis (E), and into the mesophyll (M). PA-GFP (green) is located both on the leaf surface and between the epidermis and the mesophyll cells. GFP (green) was excited using a 488 nm laser to yield maximal emission at 500 nm, chloroplasts (purple) were excited using a 488 nm laser to yield maximal emission at 700 nm, and propidium iodide stain (red) was excited using a 514 nm laser to yield maximal emission at 610 nm. We separated the green laser (488 nm) emission to distinguish between chloroplasts (700 nm) and the PA-GFP isolate (500 nm).

Confocal analysis of *Arabidopsis* plant treated with a green fluorescent protein (GFP)-tagged *P. aphidis* isolate demonstrated how *P. aphidis* can penetrate into the cell through open stomata and establish within plant tissues (Fig. 3). We could further demonstrate that following penetration, *P. aphidis* establishment within plant tissue occurred at the epidermal layer above the mesophyll cells (Fig. 4 and Fig. S2).

### *P. aphidis* activates MAMP-dependent responses in a hormone-independent matter.

**FIG 5:**
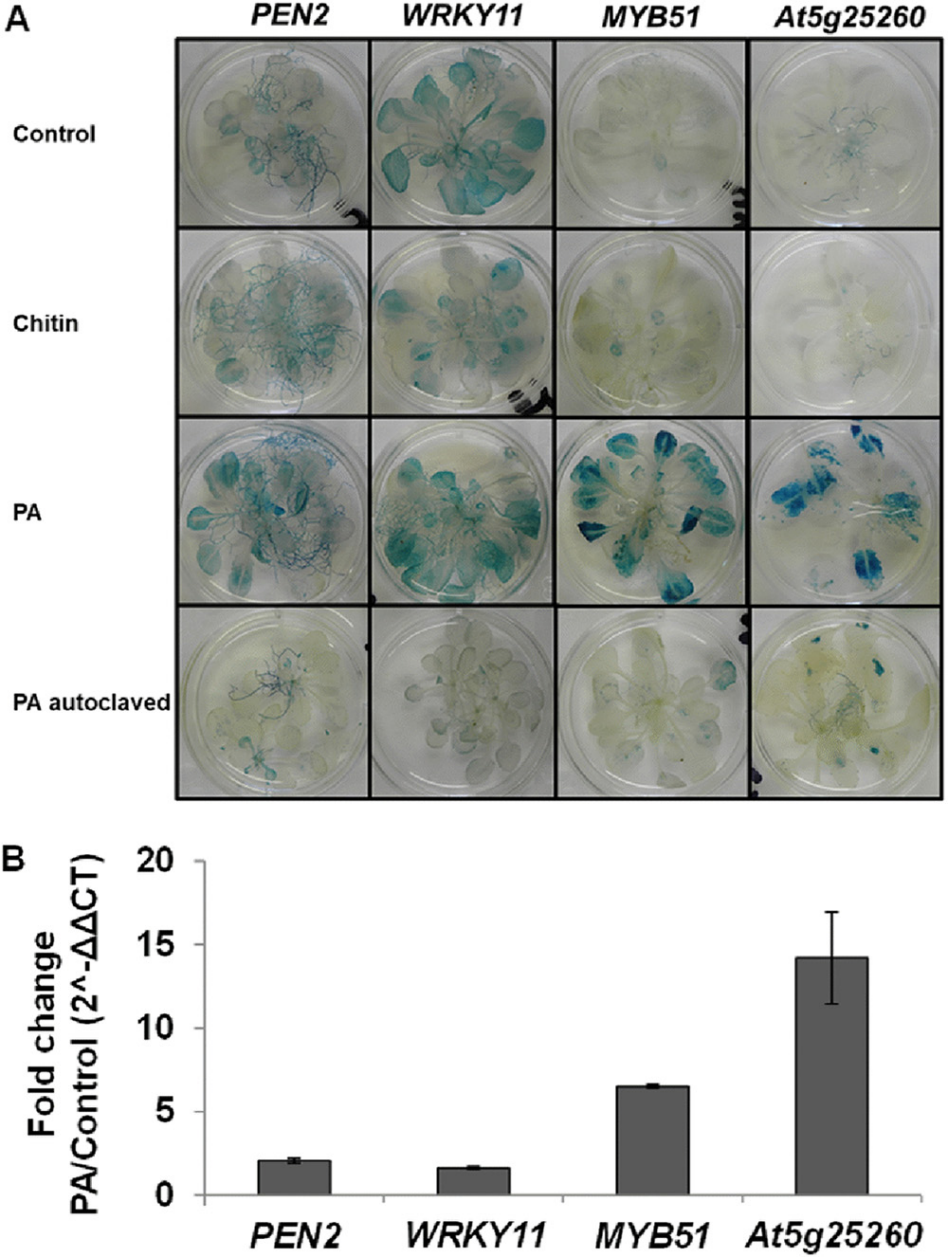
MAMP-triggered gene activation. (A) *Arabidopsis* plants carrying PEN2pro:GUS, WRKY11pro:GUS, MYB51pro:GUS, or AT5G25260pro:GUS; reporter constructs were treated with *P. aphidis* (PA; 10^8^ spores/mL), 100 µg/mL of chitin, or water (control) 4 days prior to GUS staining. Pictures represent one of three independent repeats that yielded similar results. (B) Real-time PCR analysis of the *PEN2*, *WRKY11*, *MYB51*, and At*5g25260* genes 4 days after treatment of *Arabidopsis* plants with 10^8^ spores/mL *P. aphidis* (PA). The control gene was PTB1. Averages ± the standard error from three independent experiments (*n* = 3 to 5) are presented.

We considered whether *P. aphidis* can trigger the expression of known MAMP-related genes (i.e., *PEN2*, *WRKY11*, *MYB51*, and *At5g25260*) upon establishment on *Arabidopsis* plants. We used a promoter:GUS-transgenic line assay in which upregulation of MAMPs can be detected by GUS expression (26). Figure 5A shows that the application of live *P. aphidis* onto plants resulted in significant GUS expression in leaves for all the marker genes, relative to controls. In contrast, chitin treatment activated only *WRKY11* and *PEN2*. Gene activations were also verified by quantitative real-time PCR (RT-PCR) analysis, demonstrating that the genes most activated above basal levels were *MYB51* and *At5g25260* (Fig. 5B). We could also demonstrate that such gene activation by *P. aphidis* was independent of hormonal pathways, using GUS staining of plants containing mutations in genes encoding components involved in hormone signaling via the SA (*npr1-1*), JA (*coi1*), or ethylene pathways (*ein2* or *sid2*) (Fig. S3).

### *P. aphidis* suppresses MAMP-induced callose deposition in leaves.

**FIG 6:**
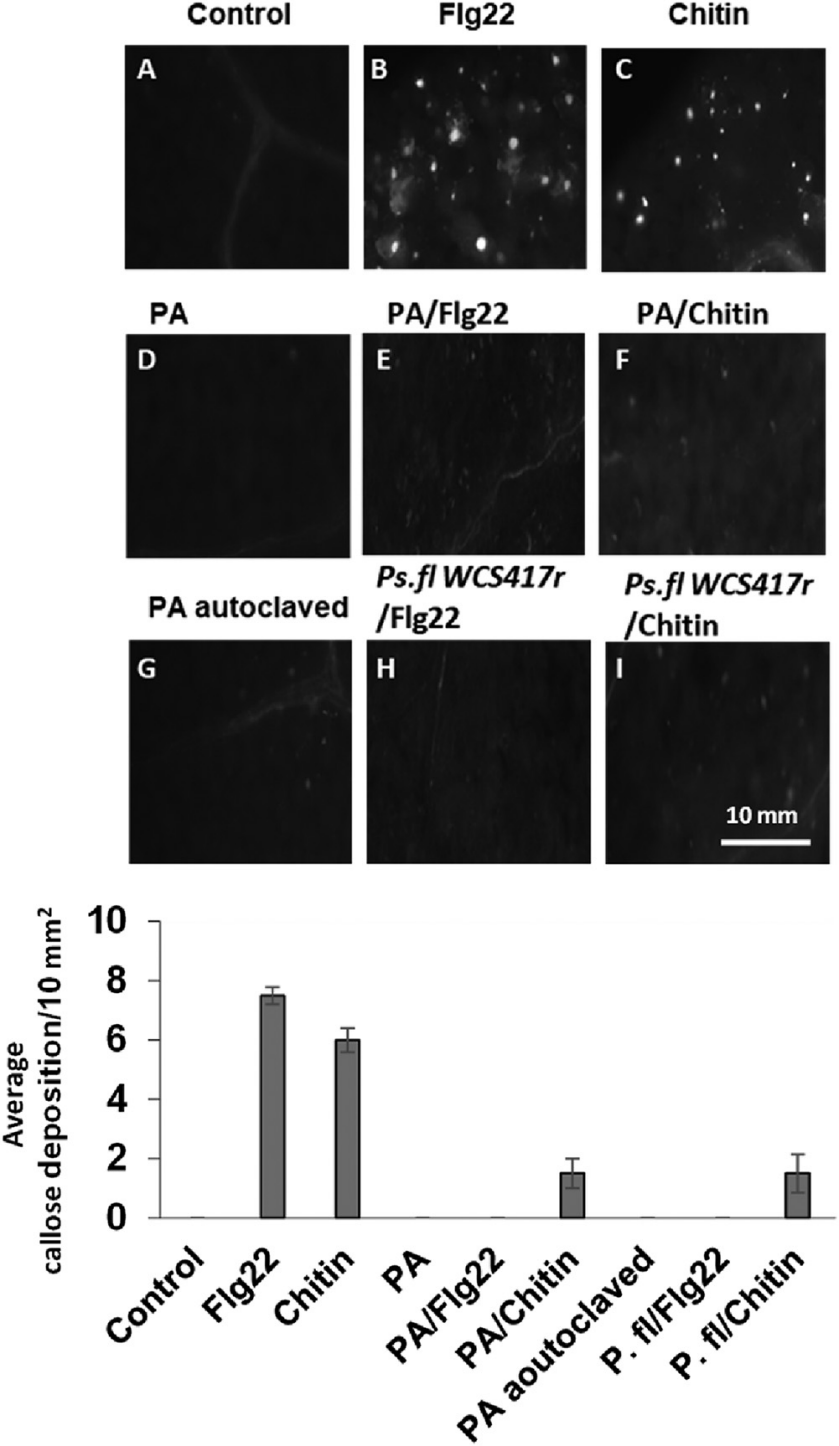
Callose deposition on *Arabidopsis* plants. Fluorescence microscopy analysis of *Arabidopsis* leaves 18 h after treatment with *P. aphidis* (PA) (10^8^ spores/mL), autoclaved PA, Flg22 (1 µM), or chitin (500 µg/mL). Plants are also treated with PA or P. fluorescens (*Ps. fl*) (10^8^ CFU/mL), 12 h prior to Flg22 or chitin treatment. Quantification of average callose deposition after different treatments is represented below as means+/−SE, n = 4. Pictures represent one of at least three independent experiments with similar results.

To study further MAMPs signaling activation by *P. aphidis*, we tested whether *P. aphidis*, normally capable of inducing systemic resistance and MAMP-triggered genes, activated callose deposition in leaves. Intriguingly, inoculation with live *P. aphidis* did not activate such callose deposition (Fig. 6D). To test the hypothesis that *P. aphidis* actively suppresses MAMPs responses in leaves, seedlings were preinoculated with *P. aphidis* prior to Flg22 or chitin treatment that induces callose deposition (Fig. 6B and C). As demonstrated in Fig. 6E and F, *P. aphidis* suppressed Flg22- and chitin-elicited activation of callose deposition in leaves, similar to Pseudomonas fluorescens (Fig. 6H and I).

### *P. aphidis* callose deposition suppression is partially dependent on SA and JA.

**FIG 7:**
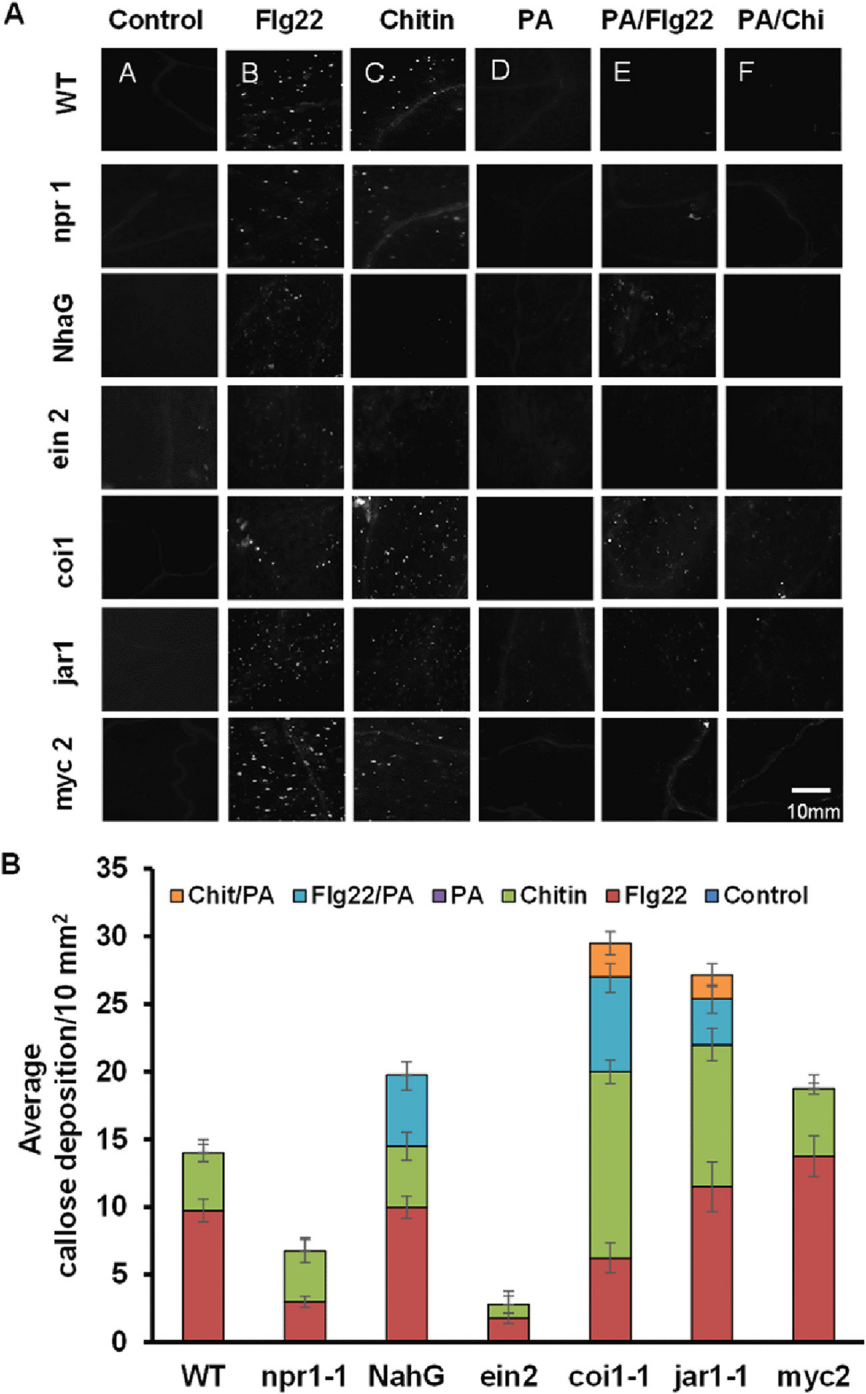
Callose deposition on *Arabidopsis* mutants. (A) Fluorescence microscopy analysis of leaves from Arabidopsis WT and mutants *npr1, NahG, ein2, coi1-1, jar1-1*, and *myc2/jin1*(*myc2*) 18 h after treatment with *P. aphidis* (PA; 10^8^ spores/mL), Flg22 (1 µM), or chitin (500 µg/mL) or treatment with *P. aphidis* 12 h prior to Flg22 or chitin treatment. (B) Quantification of average callose deposition after different treatments in WT and mutants is represented as mean+/−SE, n = 4. Pictures represent one of at least three independent experiments with similar results.

Using hormone signaling mutants, we noted that callose deposition suppression is still active in *myc2/jin1*, *npr1*, and *ein2* mutants, suggesting such suppression to be independent of NPR1, EIN2, and MYC2/JIN1 (Fig. 7). Furthermore, callose deposition suppression was suppressed only partially in *NahG*, *coi1*, and *jar1* backgrounds, suggesting that suppression is partially dependent on SA and JA signaling (Fig. 7).

## DISCUSSION

Plant-associated beneficial microorganisms, either within plant tissues as endophytes or on plant tissue surfaces as epiphytes, are important for plant nutrition, healthy status, and defense. *P. aphidis* is well established as an epiphytic yeast-like organism that protects plants from their pathogens via several modes of actions, including antibiosis parasitism and induced resistance (58–62). Our recent data demonstrated that the epiphytic *P. aphidis* can also penetrate plant tissues via openings in the stomata apparatus (Fig. 1 and 3) and establish within plant tissues in close proximity to the epidermal layer and above the chloroplast-containing mesophyll layer (Fig. 4 and Fig. S2). We also demonstrated the ability of *P. aphidis* cells to secrete cellulase and cutinase (Fig. 2), which may help *P. aphidis* degrade the cuticle and cell wall for penetration, or alternatively, for emergence back to the plant surface (Fig. 1). One can also speculate that these degrading enzymes are used by *P. aphidis* during the saprophytic phase on dead plant tissue (50). It can also be assumed that *P. aphidis* may degrade a small portion of the cuticle to settle on/within and then grip plant tissue to support its stability on the plant surface, as suggested by the fingerprinting shown in Fig. 1D. Even though endophytic and epiphytic lifestyles involve different sets of adaptations, there is some evidence for some microorganisms being both endo- and epiphyte or for endophytes that present also an epiphytic phase. For example, it was reported that the biocontrol fungus Beauveria bassiana can be naturally found as epiphytes, endophytes, and rhizosphere colonizers (63–65). Some isolates were reported to directly penetrate into plant tissue (66, 67), whereas others require wounded tissue (68) or enter through stomata (69). Another example comes with lactic acid bacteria, such as Lactobacillus plantarum, which is found as epiphyte and endophyte populations on plants (70, 71). Some *Trichoderma* species, such as T. asperellum, Trichoderma virens, and Hypocrea lixii, were isolated from both within and on the surface of banana roots (72). Additionally, it is now thought that most, if not all, pathogenic *Botryosphaeriaceae* might experience an endophytic latent phase before they infect their plant host (73). The epiphytic yeast-like fungus *P. aphidis* addressed in the current study can also be found as an endophyte, although one can also speculate that *P. aphidis* undergoes a latent phase as an endophyte that can emerge to surface only when conditions, such as nutrient availability, are optimal, either from infected plant tissue or from pathogens. Our data demonstrating the proliferation of *P. aphidis* mainly on infected tissue support this assumption (59).

In this study, we further showed that *P. aphidis* is recognized by the plant and activates the MAMP-associated genes *WRKY11*, *PEN2*, and especially *MYB51* and At*5G25260* in a manner independent of the SA-, JA-, or ET-signaling pathways (Fig. 5 and Fig. S3). Denoux et al. (74), Millet et al. (26), and Jacobs et al. (75) also showed that MAMPs treatment upregulated the expression of these defense-related genes. Nevertheless, we demonstrated that *P. aphidis* does not initiate MAMP-triggered callose deposition and could suppress MAMP-induced callose deposition by Flg22 and chitin in *Arabidopsis* plants (Fig. 6). Previous studies also showed that beneficial microorganisms and pathogens suppress Flg22-induced callose deposition in plants by secretion of effectors or other molecules (26, 76, 77). The suppression of MAMPs signaling by *P. aphidis* in leaves is partially dependent on the SA and on JA-signaling components JAR1-1 and E3 ubiquitin ligase COI1-1 (Fig. 7A) but not on NPR1, MYC2/JIN1 or EIN2 (Fig. 7B). Other beneficial microorganisms, such as the growth-promoting bacteria P. fluorescens or the beneficial root-colonizer fungus *Piriformospora indica*, can also suppress Flg22-triggered defense in a JA-dependent manner (26, 75). Nevertheless, the beneficial bacteria Bacillus subtilis does require JIN1/MYC2 in addition to JAR1 (77). Overall, these findings suggested that MAMPs suppression might correspond to a beneficial adapted strategy for their colonization and interactions with the host plant (78).

Finally, we demonstrated here that *P. aphidis* can either establish onto plant surfaces or enter plant tissues via stomata and establish within the plant. Such establishment appears to be possible after suppression of MAMP-triggered callose deposition. Other components involved in *P. aphidis* MAMPs suppression and the transition and relationship between *P. aphidis* endophytic and epiphytic phases still need to be studied and further evaluated.

## MATERIALS AND METHODS

### Plant materials.

The *Arabidopsis* GUS reporter lines MYB51pro:GUS, PEN2pro:GUS, WRKY11pro:GUS, and AT5G25260pro:GUS were kindly provided by the Ausubel lab (Harvard University) in the wild-type (WT) Columbia-0 (Col-0) or the *ein2-1*, *sid2*, *npr1-1*, or *coi1-1* mutant backgrounds (26). Following mutants and transgenic plants were also used: *NahG* (23), *npr1-1* (79), *coi1* (80), *jar1-1* (81), *ein2-1* (82), and *myc2/jin1*.

### Plant propagation.

Arabidopsis thaliana WT Col-0 and mutant seeds were sterilized in 70% ethanol for 5 min, rehydrated with 100% ethanol for 1 min, and left to dry. Seeds were germinated in petri dishes (140 by 20 cm), with each plate containing up to 10 seeds in 50 mL seedling Murashige and Skoog (MS) growth medium agar (83) and were sealed with a microporous material. *Arabidopsis* seeds were vernalized at 4°C for 2 to 3 days before being placed in a growth chamber. The seedlings were grown for 10 to 24 days at 22°C in a plant growth chamber under 8 h of light provided by a fluorescent lamp (36W/840, LUMILUX, Cool White, Osram, Munich, Germany).

### Microorganism culturing and application.

*P. aphidis* isolate L12 was maintained in solid culture on potato dextrose agar (PDA, Difco) at 26°C and transferred to fresh medium monthly. Liquid cultures were maintained in potato dextrose broth (PDB, Difco) for 3 to 7 days at 26°C on a rotary shaker set at 150 rpm. We obtained 10^8^ cells/mL after 3 days in liquid culture. Cells were centrifuged, suspended in water or MS growth medium, and applied to plants.

GFP-tagged *P. aphidis* was maintained on PDA or PDB (Difco) with 70 mg/L hygromycin B solution (TOKU-E, Bellingham, WA) at 26°C. Liquid cultures were maintained on a rotary shaker at 150 rpm. We obtained 10^8^ cells/mL after 3 days in liquid culture, which we spread (3 to 4 µL) onto the center of each leaf for induction of plant leaves.

Pseudomonas fluorescens WCS417r was maintained on King’s B medium supplemented with 50 μg/mL rifampicin (Ducefa, Haarlem, the Netherlands) at 28°C on a rotary shaker at 150 rpm. P. fluorescens was applied onto plants as reported elsewhere (26).

### Growth on cellulose or cutins.

Growth on cellulose requires cellulase activity. To assay for such cellulase activity, tap water agar (TWA) was covered with a cellulose membrane and inoculated with 4 µL of *P. aphidis* (10^8^ cells/mL) applied at the plate center. Number of cells was recorded 7 days post inoculation. To assay for cutinase activity, TWA was supplemented with 1% TCL (a cutin analogue) suspended in 100% acetone or with 0.25% acetone. Plates were inoculated with 4 µL of *P. aphidis* (10^8^ cells/mL) applied at the center of the plate. Plates were recorder 7 days post inoculation.

### Elicitor and microorganism treatments.

*Arabidopsis* WT and mutant seedlings (10 to 24 days old) grown in MS growth medium or on MS growth medium agar plates were treated with *P. aphidis* (1 × 10^8^ spores/mL), P. fluorescens (optical density at 600 nm [OD_600_] of 0.04), 1 µM Flg22 (AnSpec, Thornleigh, Australia), or 100 to 500 µg/mL chitin (Sigma, St. Louis, MO), as stated, for 18 h to 4 days before analysis. Chitin solution was prepared as described previously (60).

### GUS histochemical assay.

GUS reporter lines seedlings (24 day-old) grown on MS agar plates were transferred to 6-well microplates 18 h after treatment for GUS staining, performed as described previously (26). Briefly, 2 mL of GUS substrate solution {125 mM sodium phosphate [pH 7], 1.25 mM EDTA, 1.25 mM K_4_[Fe(CN)_6_], 1.25 mM K_3_[Fe(CN)_6_], 0.5 mM X-Gluc, and 1.25% Triton X-100} was poured into each well. The plants were vacuum-infiltrated for 10 min and then incubated at 37°C overnight, covered with aluminum foil. Tissues were destained with 100% ethanol overnight and placed in 70% ethanol before digital pictures were taken.

### Callose staining and analysis.

Following treatment, 10-day-old seedlings grown in MS medium in 12-well microtiter plates were fixed in a 3:1 ethanol:acetic acid solution for several hours. The fixative was changed several times to ensure both thorough fixing and clearing of the tissues, which is essential for good callose detection in the leaves. The seedlings were rehydrated in 70% ethanol for 2 h, 50% ethanol for an additional 2 h, and water overnight. After two or three water washes, the seedlings were treated with 10% NaOH and held at 37°C for 1 to 2 h to render the tissues transparent. After three or four water washes, the seedlings were incubated in 150 mM K_2_HPO_4_ (pH 9.5) and 0.01% aniline blue (Sigma-Aldrich) for several hours. The leaves were mounted on slides with 50% glycerol, and callose was observed immediately using a fluorescence microscope (Nikon Eclipse 80i) under UV light (excitation, 350 nm; emission, 420 nm). Pictures were taken with a Nikon DS-oiMc camera and analyzed by NIS elements BR3.10 software. For callose counting, pictures from three independent biological repeats were used, with callose being counted in four areas of each picture.

### RNA isolation and qRT-PCR analysis.

Total RNA was isolated from untreated *Arabidopsis* plants and from plants 24 to 72 h posttreatment with 10^8^ *P. aphidis* spores/mL using a Qiagen RNeasy kit (Invitrogen, San Diego, CA) according to the manufacturer’s instructions or using the LogSpin method (84). DNase treatment was performed on Qiagen columns according to the manufacturer’s instructions (Invitrogen). Total RNA (1 μg) was reverse-transcribed with an EZ-First strand cDNA synthesis kit (Biological Industries, Israel). qRT-PCR was performed with SYBR master mix and a StepOne real-time PCR machine (Applied Biosystems, Foster City, CA). The thermal cycling program was as follows: 95°C for 20 sec, 40 cycles of 95°C for 3 sec, and 60°C for 30 sec. Relative fold changes in gene expression normalized to At*EF1α* levels in samples from treated versus untreated leaves were calculated by the 2^−ΔΔCt^ method. The primer sequences used were AtPTB1F′ GATCTGAATGTTAAGGCTTTTAGCG, AtPTB1R′ GGCTTAGATCAGGAAGTGTATAGTCTCTG, WRKY11F′ CCACCGTCTAGTGTAACACTCGAT, WRKY11R′ TGCAACGGAGCAGAAGCAAGGAA, MYB51F′ ACAAATGGTCTGCTATAGCT, MYB51R′ CTTGTGTGTAACTGGATCAA, AT5G25260F′ TCGTGTTCTCGCTGCTTCCA, AT5G25260R′ GGCACGTCAACGAGCTGTTT, PEN2F′ TAACATGCTTCTAGCGCACGCAG, PEN2R′ CATCTGGATCACTCGGATCATATG.

### Confocal analysis.

*Arabidopsis* leaf samples for confocal imaging were taken 2 to 7 days after application of *P. aphidis*. Leaves were infiltrated with 100 mg/mL propidium iodide and placed into an SPL coverglass-bottom confocal dish (35 by 10 mm) in 50% glycerol. Tissues sample were observed with a TCS SP8 confocal laser scanning microscope (Leica) and GFP was excited using a 488 nm laser to obtain maximum emission 500 nm, while chloroplasts were excited using a 488 nm laser to obtain maximum emission at 700 nm. To distinguish between chloroplast autofluorescence and GFP signals, propidium iodide was excited using a 514 nm laser to obtain maximum emission at 610 nm, using ×10 NA or ×40 water NA 1.1 objective lenses. Images were analyzed with the LAF AF computer program (Leica), using an image resolution of 1,024 by 1,024. The experiment was repeated three times.

### Statistical analysis.

*t* tests were performed only when the data were normally distributed and sample variances were equal. Otherwise, a Mann-Whitney rank sum test was performed. Significance was accepted at a *P* value of <0.05 and is noted in figure or table legends. All experiments shown are representative of at least three independent repeats presenting the same pattern of results.
