# Supplementary material for: Pseudozyma aphidis Suppresses Microbe-Associated Molecular Pattern (MAMP)-Triggered Callose Deposition and Can Penetrate Leaf Tissue
Source: Microbiol Spectr. 2022 Mar 2;10(2):e02638-21. doi: 10.1128/spectrum.02638-21 (PMC8941903; doi:10.1128/spectrum.02638-21)
Supplement: SUPPLEMENTAL FILE 1 — Supplemental material. Download SPECTRUM02638-21_Supp_1_seq2.pdf, PDF file, 0.2 MB [file spectrum02638-21_supp_1_seq2.pdf]

Supplementary

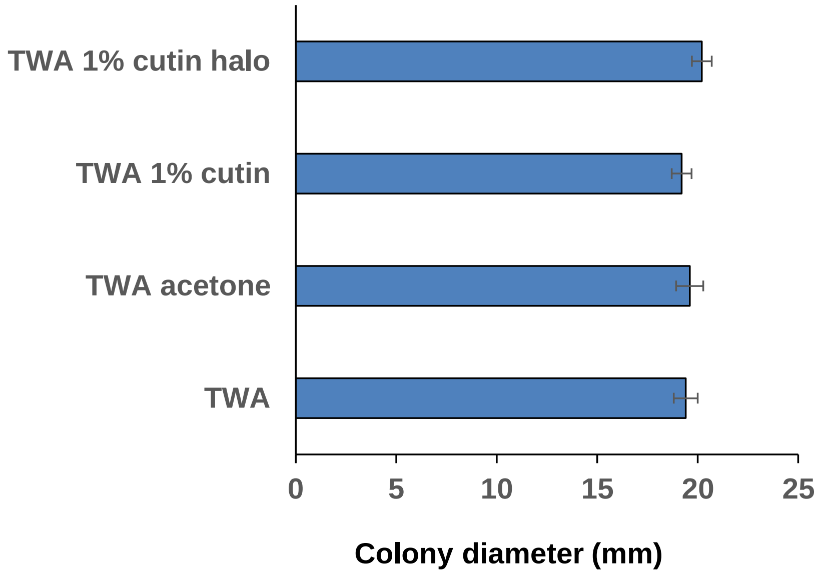

Fig S1. Cutinase activity in *P. aphidis*. *P. aphidis* grown on TWA supplemented with 1% PCL, TWA supplemented with 0.25% acetone or TWA as a control. Colony diameters were recorded four days post-inoculation with 4 µl of 10^8 cells/ml *P. aphidis*.

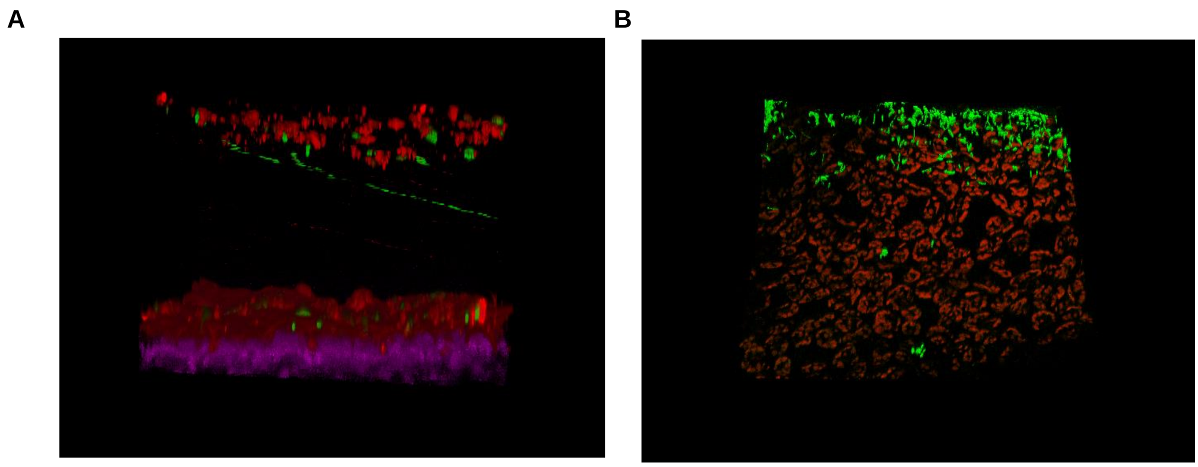

Fig. S2. *P. aphidis* localization inside Arabidopsis cells. Confocal microscopy analysis of *Arabidopsis thaliana* leaves three days post-treatment with GFP-tagged *P. aphidis* (PA-GFP). (A) The plant leaf Z stack merged view of all laser channels, where green is PA-GFP, purple

is chloroplast auto-fluorescence and red is the propidium iodide stain that outlines the epidermis and stains *P. aphidis* nuclei. PA is circled. (B) GFP (green) and chloroplasts autofluorescence (red) Z-stacks were merged top to bottom. GFP was excited using a 488 nm laser to yield maximal emission at 500 nm, chloroplasts were excited using a 488 nm laser to yield maximal emission at 700 nm and propidium iodide stain was excited using a 514 nm laser to yield maximal emission at 610 nm. We separated the green laser (488 nm) emission to distinguish between chloroplasts (700 nm) and the PA-GFP isolate (500 nm).

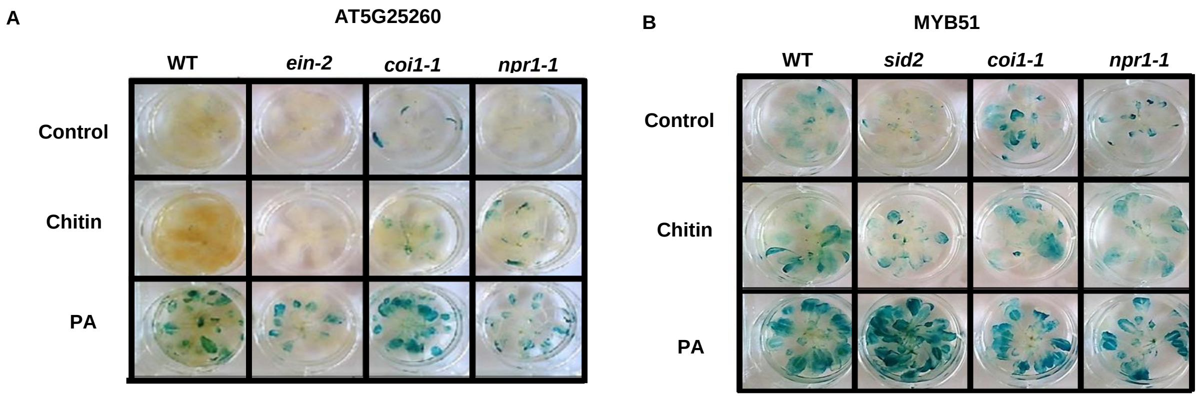

Fig. S3. MAMP-triggered gene activation in Arabidopsis mutants. Transgenic plants carrying MYB51pro:GUS (A) or AT5G25260pro:GUS (B) reporter constructs against the wild type background or *ein-2 or sid2*, *coi1-1* and *npr1-1* or mutant backgrounds were treated with *P. aphidis* (PA; 10^8 spores/ml), 100 µg/ml of chitin or water (control) for four days prior to GUS staining. Pictures represent one of three independent experiments with similar results.
